# Use of Selected Recommended Clinical Preventive Services — Behavioral Risk Factor Surveillance System, United States, 2018

**DOI:** 10.15585/mmwr.mm7013a1

**Published:** 2021-04-02

**Authors:** Suhang Song, Allison White, James E. Kucik

**Affiliations:** ^1^Policy Research, Analysis, and Development Office, Office of the Associate Director for Policy and Strategy, CDC; ^2^The Oak Ridge Institute for Science and Education, Oak Ridge, Tennessee.

Clinical preventive services play an important role in preventing deaths, and Healthy People 2020 has set national goals for using clinical preventive services to improve population health (*1*). The Patient Protection and Affordable Care Act (ACA) requires many health plans to cover certain recommended clinical preventive services without cost-sharing when provided in-network (covered clinical preventive services).* To ascertain prevalence of the use of selected recommended clinical preventive services among persons aged ≥18 years, CDC analyzed data from the 2018 Behavioral Risk Factor Surveillance System (BRFSS), a state-based annual nationwide survey conducted via landline and mobile phones in the United States, for 10 clinical preventive services covered in-network with no cost-sharing pursuant to the ACA. The weighted prevalence of colon, cervical, and breast cancer screening, pneumococcal and tetanus vaccination, and diabetes screening ranged from 66.0% to 79.2%; the prevalence of the other four clinical preventive services were <50%: 16.5% for human papillomavirus (HPV) vaccination, 26.6% for zoster (shingles) vaccination, 33.2% for influenza vaccination, and 45.8% for HIV testing. Prevalence of HIV testing had the widest variation (3.1-fold differences) across states among the 10 services included in this report. The prevalence of use of clinical preventive services varied by insurance status, income level, and rurality, findings that are consistent with previous studies (*2*–*6*). The use of nine of the 10 services examined was lower among the uninsured, those with lower income, and those living in rural communities. Among those factors examined, insurance status was the dominant factor strongly associated with use of clinical preventive services, followed by income-level and rurality. Understanding factors influencing use of recommended clinical preventive services can potentially help decision makers better identify policies to increase their use including strategies to increase insurance coverage. 

Six of the 10 recommended clinical preventive services that health plans are required to cover without cost-sharing were included in the 2018 BRFSS core questionnaire, which was used by all 50 states, the District of Columbia (DC), Guam, and Puerto Rico; these include colon, cervical, and breast cancer screening; HIV testing; and pneumococcal and influenza vaccination. The other four services were included in the optional modules, which are asked by some states; these include diabetes screening (asked by 28 states, DC, Guam, and Puerto Rico), HPV vaccination (asked by eight states), shingles vaccination (asked by four states), and tetanus vaccination (asked by four states).^†^ Survey participants were classified as having used a clinical preventive service if they reported using a clinical preventive service as recommended at the time of interview. Because of changes over time to recommendations and to policies and practices that affect use of clinical preventive services, continued monitoring of their use could offer decision makers updated information for achieving public health goals.

In the 2018 BRFSS, the median survey response rate was 49.9% with a sample size of 437,436 adults aged ≥18 years. Participants were considered uninsured if they didn’t have any health care coverage at the time of the interview. Federal poverty level (FPL) was calculated by using the number of adults, the number of children, and the midpoint income value of the categorical household income level (*7*). Persons with household income ≤138% of FPL as defined by the 2017 FPL threshold were categorized as lower income. BRFSS uses the 2013 CDC National Center for Health Statistics’ Urban-Rural Classification Scheme for Counties: urban counties are those coded as all four metropolitan categories plus micropolitan; rural counties are those coded as noncore.^§^ Weighted utilization prevalence and 95% confidence intervals (CIs) are presented. Generalized linear modeling was used to estimate prevalence ratios (PRs) and 95% CIs for the differences in use of clinical preventive services between persons in three categories: 1) insured versus uninsured, 2) higher versus lower income, and 3) rural versus urban residence. Subgroups were generated representing the interaction of these three variables, which resulted in eight insurance-income-residence combinations. Generalized linear modeling was also used to compare use of clinical preventive services use in each subgroup using STATA/MP (version 16; StataCorp), adjusted by age, sex, race/ethnicity, education, marital status, self-reported health status, and state.

Use varied across the 10 covered clinical preventive services (Table 1). The weighted prevalence of colon, cervical, and breast cancer screening, pneumococcal and tetanus vaccination, and diabetes screening ranged from 66.0% to 79.2%; the prevalence of the other four clinical preventive services were <50%, ranging from 16.5% for HPV vaccination to 45.8% for HIV testing. Being uninsured was associated with lower use of each of the 10 services, with PRs ranging from 1.03 for HIV testing to 2.99 for shingles vaccination. Persons with lower income had a lower prevalence for nine of 10 clinical preventive services compared with those with higher household incomes (eight of nine with p<0.01). In contrast, HIV testing utilization was significantly higher among those with lower income. Among those eight services, the PRs for persons with higher versus lower income ranged from 1.08 to 1.88. Persons living in rural areas used each of the recommended clinical preventive services less than those living in urban areas, with PRs for seven of these reaching statistical significance.

**TABLE 1:** Percentage of adults who received recommended clinical preventive services, by health insurance status, family income level, and rurality — Behavioral Risk Factor Surveillance System, United States, 2018

Characteristic	No. who received service, weighted % (95% CI)																			
	Colon cancer screening, age 50–75 yrs		Cervical cancer screening (women), age 21–65 yrs		Breast cancer screening (within 2 yrs) (women), age 50–74 yrs		HIV testing (ever), age 18–65 yrs		Pneumococcal vaccination (ever), age ≥65 yrs		Influenza vaccination (within 1 yr), age ≥18 yrs		Diabetes screening (within 3 yrs), age 40–70 yrs		HPV vaccination (ever), age 18–26 yrs		Zoster (shingles) vaccination (ever), age ≥50 yrs		Tetanus vaccination (within 10 yrs), age ≥19 yrs	
Total	147,965	68.4 (67.9–68.9)	106,362	79.2 (78.7–79.6)	89,409	78.7 (78.2–79.3)	113,284	45.8 (45.4–46.2)	105,829	71.0 (70.4–71.6)	164,092	33.2 (32.9–33.5)	48,719	68.8 (68.0–69.6)	527	16.5 (14.1–18.9)	6,066	26.6 (25.3–27.9)	17,390	66.0 (64.6–67.4)
**Insurance status**																				
Insured	143,667	71.0 (70.6–71.5)	97,791	81.0 (80.6–81.5)	86,525	80.4 (79.8–81.0)	100,248	46.1 (45.7–46.5)	104,463	71.6 (71.0–72.2)	158,376	35.9 (35.6–36.2)	45,840	71.2 (70.4–72.0)	471	19.1 (16.3–21.9)	5,928	28.3 (26.9–29.7)	15,668	69.4 (68.1–70.7)
Uninsured	4,035	34.1 (32.1–36.2)	8,343	66.7 (65.0–68.5)	2,719	54.2 (51.2–57.3)	12,639	44.6 (43.4–45.8)	1,138	43.9 (38.8–49.0)	5,303	13.9 (13.2–14.7)	2,781	49.8 (46.5–53.1)	54	9.8 (5.0–14.6)	122	9.5 (5.9–13.0)	1,666	51.9 (47.7–56.1)
Insured to uninsured prevalence ratio*	2.08^†^	(1.96–2.21)	1.21^†^	(1.18–1.25)	1.48^†^	(1.40–1.57)	1.03^§^	(1.01–1.06)	1.63^†^	(1.45–1.83)	2.58^†^	(2.44–2.72)	1.43^†^	(1.34–1.53)	1.95^§^	(1.17–3.25)	2.99^†^	(2.06–4.35)	1.34^†^	(1.23–1.45)
**Income level**																				
Higher income (income >138% FPL)	109,437	71.8 (71.2–72.3)	71,638	81.9 (81.3–82.4)	61,902	80.7 (80.0–81.3)	74,501	45.4 (45.0–45.9)	74,031	73.5 (72.9–74.2)	116,176	35.3 (34.9–35.7)	36,019	70.4 (69.5–71.3)	309	18.8 (15.3–22.2)	4,385	29.2 (27.6–30.8)	11,825	69.0 (67.4–70.5)
Lower income (income <138% FPL)	16,938	55.9 (54.6–57.2)	21,394	75.5 (74.4–76.5)	11,966	71.5 (70.0–72.9)	25,887	51.8 (51.0–52.7)	10,647	62.0 (60.2–63.8)	21,172	26.6 (25.9–27.3)	7,263	64.1 (61.8–66.4)	129	15.6 (10.6–20.7)	499	15.5 (12.7–18.2)	2,852	60.5 (57.0–64.1)
Higher to lower income prevalence ratio*	1.28^†^	(1.25–1.32)	1.08^†^	(1.07–1.10)	1.13^†^	(1.10–1.15)	0.88^†^	(0.86–0.89)	1.19^†^	(1.15–1.22)	1.33^†^	(1.29–1.36)	1.10^†^	(1.06–1.14)	1.20	(0.83–1.74)	1.88^†^	(1.56–2.27)	1.14^†^	(1.07–1.21)
**Rurality**																				
Urban	123,288	68.9 (68.4–69.5)	89,873	79.5 (79.0–79.9)	73,706	79.0 (78.4–79.7)	97,576	46.3 (45.9–46.7)	87,922	71.7 (71.1–72.4)	138,216	33.4 (33.1–33.7)	38,737	68.5 (67.6–69.4)	483	16.8 (14.3–19.4)	5,293	27.0 (25.6–28.5)	14,654	66.1 (64.6–67.6)
Rural	23,073	63.9 (62.6–65.1)	14,351	74.3 (73.0–75.6)	14,302	74.5 (73.0–75.9)	13,139	36.4 (35.2–37.5)	17,379	69.0 (67.5–70.5)	24,229	32.4 (31.6–33.3)	8,361	67.6 (65.7–69.6)	44	13.1 (6.6–19.5)	773	23.2 (20.3–26.1)	2,736	65.3 (61.9–68.7)
Urban to rural prevalence ratio*	1.08^†^	(1.06–1.10)	1.07^†^	(1.05–1.09)	1.06^†^	(1.04–1.08)	1.27^†^	(1.23–1.31)	1.04^†^	(1.02–1.06)	1.03^§^	(1.00–1.06)	1.01	(0.98–1.05)	1.29	(0.77–2.16)	1.17^§^	(1.02–1.34)	1.01	(0.96–1.07)

**Abbreviations:** CI= confidence interval; FPL = federal poverty level; HPV= human papillomavirus.

* Generalized linear modeling was used to identify the prevalence ratio.

^†^ p<0.01.

^§^ p<0.05.

Use of clinical preventive services varied by state (Table 2). The variation in use differed substantially by type of service, with breast and cervical cancer screenings having the least cross-state variation among the six services asked by all states. Variation across states was widest for prevalence of HIV testing and pneumococcal vaccination use (3.1-fold and 2.5-fold, respectively).

**TABLE 2:** Percentage of adults who received recommended clinical preventive services, by jurisdiction — Behavioral Risk Factor Surveillance System, United States, 2018*

Jurisdiction	% (95% CI)									
	Colon cancer screening, age 50–75 yrs	Cervical cancer screening (women), age 21–65 yrs	Breast cancer screening (within 2 yrs) (women), age 50–74 yrs	HIV testing (ever), age 18–65 yrs	Pneumococcal vaccination (ever), age ≥65 yrs	Influenza vaccination (within 1 yr), age ≥18 yrs	Diabetes screening (within 3 yrs), age 40–70 yrs	HPV vaccination (ever), age 18–26 yrs	Zoster (shingles) vaccination (ever), age ≥50 yrs	Tetanus vaccination (within 10 yrs), age ≥19 yrs
Alabama	69.8 (67.7–71.9)	79.1 (76.8–81.5)	80.2 (77.8–82.6)	45.8 (43.7–47.8)	71.7 (69.3–74.0)	67.2 (64.6–69.9)	14.6 (10.0–19.1)	NR	NR	NR
Alaska	59.6 (55.8–63.4)	76.3 (72.4–80.3)	67.3 (62.0–72.5)	45.4 (42.2–48.5)	64.2 (59.6–68.8)	33.8 (31.2–36.3)	63.5 (58.9–68.2)	NR	NR	NR
Arizona	65.8 (63.3–68.3)	76.1 (73.3–78.9)	73.1 (70.0–76.3)	45.5 (43.2–47.8)	73.7 (71.2–76.3)	30.6 (28.9–32.2)	67.9 (64.3–71.5)	NR	NR	NR
Arkansas	66.0 (63.5–68.5)	75.5 (72.8–78.3)	72.5 (69.6–75.4)	44.4 (41.8–47.0)	74.6 (72.3–76.8)	31.1 (29.2–33.0)	NR	NR	NR	NR
California	70.1 (68.2–72.0)	78.9 (77.1–80.7)	81.2 (78.8–83.5)	49.0 (47.6–50.5)	68.7 (65.8–71.6)	32.4 (31.2–33.6)	NR	NR	NR	NR
Colorado	67.8 (66.0–69.5)	76.6 (74.7–78.6)	71.1 (68.8–73.4)	41.5 (40.0–43.1)	77.1 (75.2–79.0)	36.6 (35.3–37.8)	NR	NR	NR	NR
Connecticut	74.0 (72.4–75.5)	84.5 (82.7–86.2)	82.7 (80.8–84.6)	45.8 (44.1–47.5)	71.2 (69.3–73.2)	35.0 (33.7–36.2)	NR	25.6 (20.5–30.7)	NR	NR
Delaware	72.0 (69.8–74.2)	82.4 (79.9–84.9)	83.8 (81.4–86.3)	48.6 (46.2–50.9)	72.6 (69.9–75.3)	38.2 (36.3–40.0)	NR	NR	NR	NR
DC	72.3 (69.9–74.8)	83.7 (80.8–86.5)	79.6 (76.7–82.4)	76.7 (74.4–78.9)	70.8 (68.0–73.7)	44.2 (42.1–46.3)	72.0 (68.5–75.4)	NR	NR	NR
Florida	69.6 (67.4–71.8)	79.4 (77.1–81.6)	81.2 (79.0–83.4)	52.8 (50.8–54.9)	67.2 (64.7–69.8)	30.7 (29.2–32.1)	70.6 (67.3–73.8)	NR	NR	NR
Georgia	67.4 (65.5–69.2)	80.5 (78.8–82.2)	79.8 (77.8–81.8)	52.0 (50.4–53.6)	71.0 (68.8–73.2)	29.8 (28.6–30.9)	69.4 (67.1–71.7)	NR	NR	NR
Guam	39.7 (34.6–44.8)	68.0 (62.5–73.4)	74.5 (68.0–80.9)	35.2 (31.4–39.0)	41.5 (33.6–49.5)	24.3 (21.1–27.5)	65.4 (59.1–71.8)	NR	NR	NR
Hawaii	74.3 (72.3–76.2)	83.1 (81.1–85.0)	86.9 (85.0–88.8)	35.9 (34.1–37.7)	65.4 (62.4–68.4)	33.7 (32.2–35.2)	66.5 (63.8–69.3)	16.7 (12.4–21.0)	NR	NR
Idaho	66.6 (63.6–69.6)	68.1 (64.4–71.8)	68.0 (63.9–72.1)	35.4 (32.7–38.1)	70.4 (67.3–73.6)	32.1 (30.1–34.1)	62.2 (57.9–66.4)	NR	NR	NR
Illinois	65.8 (63.4–68.2)	78.5 (76.2–80.8)	78.4 (75.4–81.5)	38.7 (36.8–40.7)	68.7 (65.7–71.7)	32.2 (30.6–33.7)	NR	NR	NR	NR
Indiana	67.1 (65.2–69.0)	78.7 (76.6–80.8)	76.4 (74.2–78.6)	41.2 (39.3–43.1)	71.8 (69.8–73.9)	28.5 (27.1–29.8)	69.6 (67.1–72.1)	NR	NR	NR
Iowa	70.9 (69.3–72.4)	79.5 (77.7–81.3)	80.7 (78.8–82.5)	30.6 (29.2–31.9)	76.1 (74.3–77.9)	40.6 (39.4–41.8)	NR	NR	NR	NR
Kansas	66.5 (65.0–68.1)	74.4 (72.5–76.3)	74.2 (72.3–76.1)	34.5 (33.0–35.9)	75.9 (74.3–77.5)	36.0 (34.9–37.2)	NR	NR	NR	NR
Kentucky	68.9 (66.4–71.4)	77.0 (74.6–79.4)	77.5 (74.7–80.4)	39.3 (37.2–41.5)	72.5 (69.6–75.4)	36.0 (34.3–37.6)	69.7 (66.7–72.7)	NR	NR	NR
Louisiana	68.5 (65.9–71.1)	82.0 (79.6–84.4)	82.9 (80.4–85.5)	48.6 (46.2–50.9)	67.9 (64.4–71.3)	26.4 (24.7–28.1)	NR	NR	NR	NR
Maine	74.9 (73.2–76.5)	82.4 (80.5–84.4)	80.8 (78.9–82.8)	39.5 (37.5–41.5)	76.7 (75.1–78.4)	32.4 (31.0–33.8)	72.3 (70.1–74.5)	NR	NR	NR
Maryland	71.5 (70.1–72.9)	81.8 (80.2–83.5)	81.1 (79.5–82.7)	55.4 (54.0–56.9)	75.3 (73.6–76.9)	39.5 (38.3–40.6)	72.9 (71.1–74.6)	NR	NR	NR
Massachusetts	75.9 (73.9–77.9)	82.9 (80.8–85.0)	86.2 (84.0–88.3)	44.6 (42.6–46.5)	72.4 (69.7–75.1)	37.1 (35.6–38.6)	NR	NR	NR	NR
Michigan	73.8 (72.2–75.4)	82.7 (81.0–84.3)	80.1 (78.1–82.1)	45.8 (44.3–47.3)	73.8 (71.7–75.9)	32.3 (31.2–33.5)	NR	NR	NR	NR
Minnesota	72.5 (71.2–73.7)	81.0 (79.7–82.3)	82.2 (80.7–83.6)	35.1 (34.0–36.2)	72.5 (71.0–74.1)	39.7 (38.8–40.6)	NR	NR	NR	NR
Mississippi	62.0 (59.7–64.4)	80.4 (78.2–82.5)	71.0 (68.2–73.9)	47.3 (45.1–49.4)	68.6 (65.9–71.3)	32.7 (31.1–34.3)	64.1 (61.2–66.9)	15.7 (10.9–20.6)	NR	57.2 (55.4–59.1)
Missouri	69.2 (66.9–71.5)	77.5 (74.8–80.3)	75.3 (72.4–78.1)	39.4 (37.2–41.7)	73.9 (71.6–76.3)	36.5 (34.8–38.2)	70.3 (67.3–73.4)	17.1 (11.7–22.5)	27.0 (25.2–28.8)	71.2 (69.3–73.0)
Montana	63.3 (60.8–65.8)	73.5 (70.4–76.5)	73.7 (70.5–77.0)	37.4 (35.2–39.6)	73.4 (70.6–76.2)	35.7 (33.9–37.4)	NR	NR	NR	NR
Nebraska	68.1 (66.5–69.7)	78.0 (76.2–79.7)	75.2 (73.2–77.3)	29.6 (28.2–31.0)	76.4 (74.8–78.1)	39.4 (38.1–40.6)	NR	NR	NR	NR
Nevada	59.9 (56.0–63.9)	77.4 (73.6–81.2)	72.3 (67.9–76.8)	47.2 (44.1–50.3)	68.1 (63.5–72.7)	32.6 (30.1–35.0)	NR	NR	NR	NR
New Hampshire	74.1 (72.1–76.1)	82.9 (80.5–85.2)	82.8 (80.6–85.1)	42.0 (39.7–44.4)	78.5 (76.4–80.6)	33.3 (31.6–35.0)	NR	NR	NR	NR
New Jersey	66.6 (62.3–70.9)	78.8 (74.4–83.2)	80.8 (76.1–85.5)	49.8 (46.3–53.3)	67.8 (61.5–74.1)	38.1 (35.3–41.0)	78.4 (73.8–83.0)	18.8 (10.5–27.1)	NR	NR
New Mexico	63.8 (61.5–66.1)	77.1 (74.7–79.4)	71.6 (68.8–74.5)	38.4 (36.4–40.4)	70.9 (68.1–73.8)	34.3 (32.7–35.9)	71.4 (68.6–74.3)	NR	NR	NR
New York	68.9 (67.5–70.4)	81.5 (80.1–82.9)	82.1 (80.4–83.9)	57.0 (55.8–58.1)	63.8 (61.6–66.0)	28.0 (27.1–28.9)	65.2 (63.4–67.0)	NR	NR	NR
North Carolina	71.0 (68.5–73.5)	80.3 (78.0–82.7)	79.3 (76.1–82.5)	52.1 (49.9–54.2)	76.2 (73.0–79.5)	41.7 (39.9–43.5)	72.0 (68.8–75.2)	NR	NR	NR
North Dakota	66.5 (64.3–68.7)	75.0 (71.9–78.1)	78.9 (76.2–81.6)	33.6 (31.4–35.8)	75.0 (72.8–77.2)	40.0 (38.2–41.9)	65.3 (62.4–68.2)	NR	NR	NR
Ohio	66.6 (64.9–68.3)	79.0 (77.1–80.8)	77.4 (75.4–79.3)	39.6 (38.0–41.3)	74.1 (72.3–75.9)	35.2 (33.9–36.4)	NR	NR	NR	NR
Oklahoma	62.1 (59.7–64.5)	73.5 (71.0–76.1)	74.2 (71.4–77.0)	36.4 (34.2–38.5)	74.8 (72.4–77.2)	38.1 (36.5–39.8)	NR	NR	NR	NR
Oregon	71.7 (69.5–73.9)	78.0 (75.7–80.3)	77.9 (75.3–80.6)	45.7 (43.8–47.6)	77.1 (74.4–79.9)	30.6 (29.1–32.1)	67.7 (64.8–70.5)	NR	NR	NR
Pennsylvania	71.3 (69.1–73.5)	77.3 (74.8–79.8)	78.6 (75.8–81.5)	41.4 (39.4–43.3)	74.7 (71.9–77.5)	40.3 (38.7–41.9)	NR	NR	NR	NR
Puerto Rico	55.7 (53.2–58.3)	81.6 (79.5–83.7)	83.5 (81.0–86.0)	61.5 (59.4–63.6)	31.1 (28.0–34.2)	25.8 (24.2–27.3)	85.5 (83.3–87.7)	NR	NR	NR
Rhode Island	75.1 (72.9–77.3)	83.9 (81.5–86.4)	87.0 (85.0–89.0)	48.4 (46.0–50.8)	74.6 (72.1–77.1)	37.1 (35.2–38.9)	NR	NR	NR	NR
South Carolina	70.3 (68.7–71.9)	78.7 (76.8–80.6)	77.2 (75.2–79.1)	44.3 (42.6–46.0)	73.4 (71.7–75.1)	35.5 (34.3–36.8)	69.7 (67.4–71.9)	NR	NR	NR
South Dakota	68.4 (65.6–71.2)	72.9 (68.9–76.9)	81.7 (78.6–84.8)	32.0 (29.4–34.6)	76.5 (73.7–79.4)	35.3 (33.2–37.4)	63.7 (59.9–67.4)	NR	NR	NR
Tennessee	68.3 (65.7–70.8)	78.9 (76.2–81.7)	76.3 (73.1–79.5)	42.5 (40.1–44.9)	74.2 (71.3–77.0)	28.6 (26.9–30.3)	69.0 (65.7–72.2)	19.8 (12.3–27.2)	24.6 (22.5–26.8)	NR
Texas	59.3 (55.9–62.8)	78.2 (75.5–81.0)	74.9 (70.4–79.5)	47.1 (44.8–49.5)	71.1 (67.2–74.9)	26.4 (24.6–28.1)	61.6 (57.2–65.9)	13.7 (9.3–18.1)	25.7 (23.2–28.2)	62.7 (60.3–65.1)
Utah	69.5 (67.7–71.3)	73.0 (71.2–74.9)	72.3 (69.8–74.8)	24.7 (23.5–25.9)	73.7 (71.6–75.9)	32.3 (31.2–33.5)	NR	NR	NR	NR
Vermont	71.2 (69.2–73.1)	78.4 (75.8–81.0)	76.4 (73.9–78.9)	44.1 (41.8–46.3)	74.5 (72.2–76.9)	37.2 (35.4–38.9)	NR	NR	NR	NR
Virginia	69.3 (67.5–71.1)	83.6 (81.9–85.3)	81.1 (79.1–83.1)	47.3 (45.6–49.0)	73.6 (71.5–75.7)	38.9 (37.6–40.3)	71.3 (69.1–73.6)	NR	30.0 (28.5–31.5)	73.5 (72.1–74.8)
Washington	70.8 (69.3–72.4)	76.3 (74.6–78.1)	74.8 (72.8–76.8)	44.9 (43.4–46.3)	77.7 (76.2–79.3)	38.4 (37.3–39.5)	67.0 (64.9–69.1)	NR	NR	NR
West Virginia	67.4 (65.3–69.5)	78.0 (75.6–80.4)	75.1 (72.3–77.9)	35.1 (33.0–37.2)	73.0 (70.6–75.4)	42.6 (40.9–44.3)	73.4 (70.7–76.0)	NR	NR	NR
Wisconsin	74.0 (71.6–76.4)	79.5 (76.9–82.0)	77.8 (74.8–80.9)	33.9 (31.6–36.1)	74.7 (71.7–77.7)	29.9 (28.1–31.6)	68.6 (65.6–71.7)	NR	NR	NR
Wyoming	57.7 (55.3–60.1)	73.2 (70.4–76.1)	68.0 (64.9–71.0)	38.0 (35.8–40.3)	69.8 (67.2–72.3)	31.0 (29.4–32.7)	60.2 (57.0–63.3)	NR	NR	NR

**Abbreviations:** CI = confidence interval; DC = District of Columbia; HPV = human papillomavirus; NR = not reported.

* 50 states, DC, Puerto Rico, and Guam were included in the analysis of cancer screenings, HIV testing, pneumococcal vaccination, and influenza vaccination; 28 states, DC, Puerto Rico, and Guam were included in the analysis of diabetes screening; eight states were included in the analysis of HPV vaccination; and four states were included in the analysis of both zoster (shingles) and tetanus vaccinations.

The highest adjusted use was observed in the insured-higher income-urban group for six of the 10 services (all but HIV testing, diabetes screening, HPV vaccination, and tetanus vaccination). Insurance status was the factor most strongly associated with use of clinical preventive services, followed by income level and rurality, respectively. Uninsured persons used seven of the 10 clinical preventive services less frequently than those with insurance, regardless of income level and rurality. Among those with insurance, use of six of the 10 services was higher among persons with higher incomes, regardless of whether they lived in rural or urban counties (Figure) (Supplementary Figure, https://stacks.cdc.gov/view/cdc/104149) (Supplementary Table 1, https://stacks.cdc.gov/view/cdc/104150).

**FIGURE:**
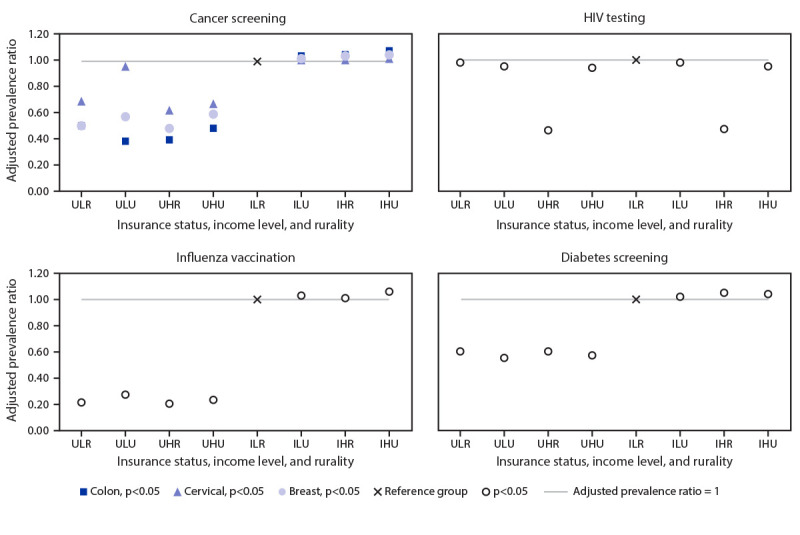
Adjusted prevalence ratios of use of selected clinical preventive services,* by health insurance status, family income level, and rurality — Behavioral Risk Factor Surveillance System, United States, 2018 **Abbreviations:** HPV= human papillomavirus; IHR = insured and higher income and rural; IHU = insured and higher income and urban; ILR = insured and lower income and rural; ILU = insured and lower income and urban; UHR = uninsured and higher income and rural; UHU = uninsured and higher income and urban; ULR = uninsured and lower income and rural; ULU = uninsured and lower income and urban. * Adjusted by age, sex (except for cervical cancer screening and breast cancer screening), race/ethnicity, education level, marital status, self-reported health status, and state. Similar findings were observed in pneumococcal, HPV, zoster (shingles), and tetanus vaccinations (panels available in Supplementary Figure, https://stacks.cdc.gov/view/cdc/104149).

## Discussion

In 2018, use of nine recommended clinical preventive services was lower among persons without insurance, those with lower income, and those living in rural communities, whereas use of HIV testing was higher among persons with lower income. Geographic variation in use of clinical preventive services existed across states. These differences varied by type of services, with variation being greatest for HIV testing use. Insurance status had the strongest association with use of clinical preventive services followed by income and rurality.

Use of nearly all recommended clinical preventive services was higher in 2018 than it was during 2011–2012 (*3*,*4*). These results were consistent with previous studies, which showed that the prevalence of use of clinical preventive services was lower among persons who were uninsured, lived in households with lower income, and lived in nonmetropolitan areas (*3*–*5*). Geographic variation was also consistent with previous studies, which suggests that state-level variation could be used to identify state- and locality-specific strategies to increase use of clinical preventive services (*6*,*8*). In addition, policies that address health insurance coverage and benefits or reduce specific barriers to care for persons with lower income or living in rural areas could potentially be effective at increasing use of clinical preventive services. The finding that use of HIV testing was higher among persons of lower income was consistent with previous studies (*2*,*3*) and might reflect the success of a testing strategy that focused HIV screening efforts in communities that are disproportionately comprised of persons of lower income (*9*). Fear and misperceptions about HIV risk and the testing process itself might be additional barriers to increasing HIV testing (*10*).

The findings in this report are subject to at least six limitations. First, the analysis was based on self-reported use data, which could be subject to recall and social desirability bias. Second, use of some services as measured by BRFSS was not entirely aligned with the recommendations; BRFSS questions, recommendations, and important distinctions are provided (Supplementary Table 2, https://stacks.cdc.gov/view/cdc/104148). Third, FPL was estimated based on the categorical income value provided by BRFSS rather than a precise estimate of household income. Fourth, whether BRFSS participants received services from in-network providers could not be determined, nor could whether survey participants were enrolled in insurance plans subject to ACA requirements to provide clinical preventive services without cost-sharing be determined (*1*). Therefore, use among the insured group was potentially underestimated compared with a sample comprised entirely of persons with ACA-compliant plans. Fifth, this is a cross-sectional study, and causal relationship cannot be determined even when relevant confounders are adequately controlled. Finally, only a limited number of states participated in BRFSS optional modules for diabetes screening and for HPV, shingles and tetanus vaccinations, and so data might not be nationally representative of prevalence, even though the results were consistent with previous studies (*3*,*4*).

As the health care policy landscape continues to shift, understanding factors associated with use of recommended clinical preventive services could help decision makers better identify policy levers to increase use of clinical preventive services. The ongoing monitoring of trends could improve understanding of how modifiable factors affect use of clinical preventive services, especially during the pandemic, because a decrease in use of routine vaccinations was observed. Although insurance status, income level, rurality, and state of residence appear to be associated with use, examining other barriers could also help better identify strategies to achieve public health goals.

**Summary**

**What is already known on this topic?**

Ongoing federal and state health reform efforts, particularly the Patient Protection and Affordable Care Act, have affected use of clinical preventive services in the United States.

**What is added by this report?**

Analysis of 2018 Behavioral Risk Factor Surveillance System data indicated use increased for selected recommended clinical preventive services; however, use of nine of the 10 services examined was lower among the uninsured, those with lower income, and those living in rural communities. Among those factors examined, insurance status had the strongest association with use of clinical preventive services, followed by income level and rurality.

**What are the implications for public health practice?**

Understanding factors influencing use of clinical preventive services can potentially help decision makers better identify policies to increase their use including strategies to increase insurance coverage.

## Footnotes

* The Patient Protection and Affordable Care Act, Pub L. No. 111–148, 124 Stat. 131, Sect. 1001 (Mar. 23, 2010). The covered clinical preventive services were recommended by the U.S. Preventive Services Task Force, the Advisory Committee on Immunization Practices, and the Health Resources and Services Administration.

† The BRFSS questionnaire has three parts: 1) the core component, 2) optional modules, and 3) state-added questions. Every state must ask the core component questions; however, the modules are optional. https://www.cdc.gov/brfss/questionnaires/index.htm

§
https://www.cdc.gov/nchs/data/series/sr_02/sr02_166.pdf
